# Implementing Asset-Based Integrated Care: A Tale of Two Localities

**DOI:** 10.5334/ijic.5621

**Published:** 2021-11-17

**Authors:** Sandhya Duggal, Robin Miller, Denise Tanner

**Affiliations:** 1 University of Birmingham, Birmingham, UK

**Keywords:** local area coordination, asset-based, integrated care, implementation

## Abstract

**Background:**

To date, few studies have examined the implementation of asset-based integrated care in the UK. This paper aims to address this gap in knowledge through examining the implementation of one model of asset-based integrated care, Local Area Coordination (LAC), within two localities in England.

**Methods:**

This paper draws upon data collected from two local authorities (site A and site B), which had both implemented LAC. Using a case study approach, qualitative data was collected from interviews with relevant stakeholders both internal and external to the local authorities. Data was analysed thematically.

**Results:**

The findings demonstrate the marked differences between the two sites’ approaches to LAC, especially in relation to: the implementation process; impact; and their collaboration with other agencies and communities.

**Discussion:**

The evidence presented in this paper demonstrates that the implementation of LAC, as with most complex service innovations, is dependent on the interplay of organisational and people-based components. In particular, successful implementation depends on maintaining a common vision of what an intervention will achieve and how it will work in practice, continual engagement with the political and organisational leaders of influence, positively addressing the anxieties of existing services and professions, and working with community groups.

## Introduction

The importance of integrated care encompassing not only ‘clinical pathways’ between professionals but also partnership working between health care and broader community agencies has long been recognised in holistic models [1,2]. This recognises that health outcomes for individuals and populations are significantly influenced by factors outside of the scope and control of direct health services. To achieve more equitable societies and counter rising health inequalities, wider social determinants including “early years’ experiences, education, economic status, employment and decent work, housing and environment” [3] must be pro-actively addressed through population health improvement [4]. Alongside better coordination of the activities and resources of formal public services, such strategies should seek to build on community assets that have been commonly over-looked or under-valued by governments [5]. These assets, such as cultural capacities, social networks and natural resources incorporate those held by individuals and families and non-government organisations which rely on charitable income and/or voluntary effort [3,6].

Integrated care policies in England have not only sought to improve the relationship between the delivery of formal health and social care services [7], but also to embed a preventative approach around wellbeing and public health. In public health, for example, the value of community connections is increasingly recognised in terms of the impact of social isolation on a range of health conditions [8]. Legislative changes have allowed for wider system changes within health and care, facilitating person and community centred approaches. For example, the Care Act 2014 requires local government to promote the integration of care and support services with health and wider partners, such as housing, where this will promote the wellbeing of adults and carers and contribute to the prevention or reduction of need for care services. Such flexibilities were explored through national innovation programmes including Integrated Care and Support Pioneers [9], Integrated Personal Commissioning [10], and the New Models of Care [11]. Asset based approaches include: peer support [12], community navigators [13], social prescribing [14], micro-enterprises [15], and Asset Based Community Development (ABCD) [16,17]. Though far from conclusive, emerging evidence indicates grounds for optimism [18].

In common with the implementation and sustainability of most complex service innovations, components that determine success of integrated care programmes are “multifactorial in nature and are characterized by a complex interplay” [19]. Implementation models highlight many of the building blocks that facilitate integrated care systems [20], but there remain knowledge gaps about the most effective way to organise and support these in practice within local contexts [21]. Goodwin (2019) describes this as the ‘black box’ of complexities, which also encompasses the lack of research at operational level [22]. Such complexities and related challenges of implementation are arguably heightened when integrated care requires partnership working between formal health and care services and community based voluntary organisations. Many local assets and knowledge are held within small and informal organisations which often lack the capacity, infrastructure, experience or confidence to work as equal partners with larger health organisations [23]. In addition, government organisations that fund community groups often produce unequal power dynamics [24], which may diminish potential opportunities for expanding or developing assets [25]. Seaton et al’s review of the factors which impact on inter-organisational health promotion collaborations concluded that it “remains unclear the extent to which each of the facilitating and constraining factors identified contribute collaborative success” [26].

This paper aims to address this gap in knowledge through reflecting on the implementation of one model of asset-based integrated care, Local Area Coordination (LAC), within two localities in England.

### Local Area Coordination

Originating in Western Australia during the 1980s, LAC originally emerged to support individuals with learning disabilities living in rural areas, offering people direct family support and access to services [27]. Over time, it extended to urban areas across Australia and an adapted version (in which there is more emphasis on the care planning role of coordinators) is a central component of the National Disability Insurance Scheme (NDIS) introduced in 2013 [28]. LAC has gained global interest and there has been engagement within the UK in particular. Scotland was the first UK nation to adopt LAC in 2009 to support people with learning disabilities [29]. After 2010, a growing number of English and Welsh local authorities introduced LAC, supported by the LAC Network [30].

LAC aims to help people and their communities to be self-supporting by transforming systems and strengthening relationships between people, communities and services [27,31]. The model functions at both systems and individual levels. At a systems level, LAC aims to move from a crisis to prevention focus, supporting the organisational shift to strengths-based capacity building to increase the range of support and services available to people. It also seeks to build connections with and add value to existing asset-based initiatives, such as social prescribing or micro enterprise. At the individual level, LAC aims to reduce individual dependence on services and help people to find non-service solutions to their social care problems. LAC emphasises the importance of building supportive personal relationships and developing better, more resourceful communities [32]. This is primarily achieved through the role of the Local Area Coordinator, whose role is to ‘walk alongside’ individuals in their communities to help them build their own vision of a good life [31]. Coordinators aim to support people to stay independent and connected with their communities, whilst finding pragmatic solutions to problems, drawing on family and community resources (see ***Table 1***). Coordinators are place-based, defined by a geographical area and work flexibly to build positive and trusting relationships with individuals, families and communities.

**Table 1:** Local Area Coordination Core Princples and Local Area Coordinator role.


Local Area Coordination – Core Principles [33]	The right to citizenship, responsibilities and opportunities The importance of valued relationships and personal networks The importance of access to relevant, timely and accessible information to inform decision making Recognising and nurturing individual, family and community gifts and assets Recognising the natural expertise and leadership of people labelled as vulnerable and their families The right to plan, choose and control supports and resources The value and complementary nature of formal services as a back up to natural supports and practical solutions

Role of a Local Area Coordinator [34]	Local Area Coordinators are expected to help people: Seek practical, non-service solutions to issues and problems wherever possible Access, navigate, coordinate and control services and support if these are required Build and maintain valued, mutually supportive relationships Understand and nurture their gifts, skills, experiences and needs Access accurate, relevant and timely information Build a positive vision and plan for the future Be part of, and actively contribute to, community life Be heard (LACs encourage self-advocacy, advocate alongside people, or advocate for people if there are no other options)


There has been a recent increase in the adoption of LAC in the UK, with 11 regions implementing LAC and 90 coordinators working across England and Wales [30], and a growing body of independent academic reviews and evaluations [35,36,37,38]. Early evaluations found that positive outcomes are largely dependent on fidelity to the design (the connected role, values, principles and practice, whole person/family/community approach) and strong, connected, contributing senior leadership [30,35]. However, evidence is stronger for positive individual and family-level outcomes, whereas community-level evidence (i.e. how activities build social capital) and broader system transformation remains tentative [35]. Our research contributes to building knowledge of factors that are key to LAC realising its potential as an enabler of community focussed integrated care.

## Methods

This paper draws upon data collected as part of a larger research study funded by the Department of Health and Social Care to explore how prevention has progressed following the Care Act 2014 [39]. In England, Local Authorities have responsibility for the planning and oversight of social care, social housing and health promotion, and coordinating multi-agency working through Health and Wellbeing Boards. The research question sought to find out what different approaches were being tried out by seven local authorities and how effective they were in; a) increasing the capability of communities, families and individuals and b) preventing, reducing or delaying the need for social care services.

The research study comprised an online national survey and a case study methodology to investigate seven local authorities who were undertaking innovative work in prevention. Within the case study sites between 2017- 2018, we undertook interviews with stakeholders, service users and carers and collected data about service-user outcomes via Quality of Life (ICECAP-A) questionnaires [40]. The research study was guided by a Lived Experience Advisory Panel with representation from the case study sites. Local community champions’ voices were also represented through their participation in interviews and focus groups as part of the data collection. Ethical permission was obtained from the Social Care Research Ethics Committee.

This paper draws upon data collected from two local authorities (site A and site B – see ***Table 2***). These two local authorities were selected for discussion in this paper as they were the only two sites that had implemented LAC. Both sites are largely urban areas on the outskirts of major cities, with deprived and increasingly diverse populations, though Site B has areas of higher affluence. In both cases, LAC was part of a strategy to move towards a more community orientated model based on encouragement to access informal networks and voluntary support before resorting to formal health and care services. However, their approach to implementation of LAC showed marked differences, as discussed in the findings section below.

**Table 2:** Site characteristics.


	SITE A	SITE B

POPULATION	167,025 (ONS, 2016)	206,674 (CENSUS, 2011)

Organisational context	LAC provided within the local authority LAC introduced as one part of transformation programme Joint involvement with CCG Clear phased development plan	LAC provided within the local authority LAC introduced as part of a Health and Wellbeing Strategy LAC dovetailed with existing services Unclear development plan

Number of localities	Introduced in a limited number of localities and expanded steadily to whole of local authority area	Piloted in five localities (out of seventeen in total)

Number of coordinators	14	5

Funding	Adult Social Care, Better Care Fund and public health	NHS Vanguard. No longer term funding identified


The findings discussed here are based on the following data collected in Sites A and B: 32 interviews and 4 focus groups (approximately 10 people in each focus group) with relevant stakeholders both internal and external to the local authorities (i.e. senior management, LAC coordinators, voluntary sector). The findings in this paper are primarily based on this data, but also on the observations and experiences gained from the wider project of the LAC services including people with lived experience. All interviews were audio recorded, transcribed and analysed using NVivo coding and thematic analysis [41].

## Results

We present key findings under three themes: implementation process; impact and collaboration with other agencies and communities. These themes were developed through the data analysis of the interviews and focus groups.

### Implementation process

Both sites aspired to introduce the same model of integrated community-based support with much similarity regarding core elements of their implementation process. LAC was framed within a wider vision to achieve more preventative care based on principles of co-production and partnership. Both drew on advice from the national LAC network and gained peer support and insights from other areas. The two sites decided to employ their coordinators within the public sector rather than commission the service from the private or voluntary sectors. Communities were involved in the recruitment of coordinators and they were selected on the basis of values, ability to engage and local knowledge rather than a professional qualification. Coordinators were expected to locate themselves predominantly within community bases and spend time developing relationships with public and voluntary organisations. Implementation began in both within a limited number of localities with the aspiration that it would in time be introduced across the local authority, although it was only Site A that was able to achieve this aspiration.

Despite these similarities, the implementation in Site A was undoubtedly more successful. Unlike Site B, LAC became available to the whole population as a core element of local provision, not only by social services but also by other sectors including health, housing, and community-based organisations. There was a conviction from the outset from key decision makers in Site A that LAC should become mainstream activity. The initial limited introduction was due to not all the necessary funding being secured at the beginning, rather than any question about whether this should happen, and the first areas were seen as generating insights for wider implementation. In Site B, five areas were selected to test out LAC. It was quickly emphasised that long-term funding and wider adoption of the model were dependent on a demonstration of financial efficiency. Whereas in Site A, LAC was assumed to be effective and the issue was how best to implement it, in Site B LAC had to prove its case in a challenging, if not unrealistic, timescale. This was reflected in the type of funding that was deployed – in Site A much of the funding came from mainstream budgets, whereas in Site B the pilot was supported through a time-limited innovation grant from central government. This meant that within Site B there was an anxiety about long-term sustainability, which was heightened when funding for a similar local service (care navigation) was withdrawn. Site A also saw the importance of understanding impact and sharing this with local stakeholders but this was from a position of much greater confidence and security.

Leadership also played a major role in shaping these different assumptions of the value of LAC. In Site B, LAC was introduced by the most senior social services director who left during the period of the pilot with their successor not favouring the approach. There was also turnover at this level within Site A, but influential operational managers who championed LAC from the outset remained within the local authority. They secured support from new senior directors and worked to promote its effectiveness with local politicians. Engagement in national networks and good practice events helped to consolidate its local standing alongside sharing learning with other areas. Beyond such stakeholder diplomacy, the leaders of LAC within Site A also sought to embed a culture in which the coordinators (and their service manager) had considerable autonomy to respond creatively to local needs. They felt positively about what was being achieved and were keen to promote this. In Site B, there was considerable criticism of the operational management style and a perceived lack of support for the coordinators from senior leaders. These contrasting experiences are illustrated here:

“We have always somehow found the time to kind of be a sort of modern day evangelist. So we’ve gone around a lot, we’ve worked with a lot of other local authorities, we shared our learning, we’ve always responded positively to any introduction to come and tell people about what we’re doing. … if you get some people who have respect and a reputation on board and they start to say good things about you, it makes life easier.” (Site A: LA Manager)

“I was telling people within the Council about them (coordinators) that didn’t even know that they existed, … that should be the role of the manager in my opinion … I get the feeling … they were just left to their own devices … I think it’s been really disappointing because I think the people at the top haven’t led it at all.” (Site B: Community Member)

The Sites also differed in their success in, and perhaps commitment to, engaging with local citizens in the development and management of LAC. In Site A, there were efforts to develop and maintain a stakeholder group of people who had accessed LAC to provide challenge and support to the running of the service. Although this presented some logistical difficulties in practice, an informal network of people with direct experience was established and there were plans to involve the user network in more strategic discussions in the future. In contrast, there was criticism from some community groups in Site B about the perceived minimal and tokenistic attempts to involve people with lived experience in the planning and operation of LAC:

“It would have been nicer to be involved in the actual coproduction of the solution in the first place. The community weren’t involved in that. It was, ‘We’re going to have this scheme. Now we want you involved’. It was that kind of process, rather than saying, ‘What’s the best scheme to work in this area and how do we work through that appropriately?’ and ‘How do we make sure that the community remain engaged in it?”. (Site B: Local community organisation representative)

### Impact

The two local authorities had different expectations of LAC. Whereas Site A was anticipating that it would deliver increased value from the same service costs, Site B was expecting it to demonstrate cost savings. Both sites found it problematic to demonstrate the impact of LAC in terms of increased value (Site A) and reduced costs (Site B). Neither site had a structured process for gathering and monitoring outcomes for the LAC service. This was in part due to the challenges of measuring preventative impacts and being able to attribute beneficial outcomes to LAC.

Both sites approached monitoring and evaluation activities differently. In Site A, early intervention reports played an influential role in making the case for LAC. Initially there was considerable scepticism within the wider LA and partners around the potential benefits of LAC. However, as feedback and evidence of its benefited emerged, LAC became increasingly valued by strategic leaders:

“I absolutely feel we’ve proved the point now, so the fact we’ve been able to sustain it and build it – at the beginning there was real scepticism about LAC. It was something that could’ve been dropped easily, it was nice to do, but not essential. Everywhere in the council I go now people talk about LAC … so the reward is huge.” (Site A, LA Manager)

Early intervention reports drew on a range of outcome data by combining quantitative data alongside people’s individual stories about the benefits of the service. These reports became an important part of the sustainability of the programme in the longer term, with senior leaders fully aware of the importance of demonstrating beneficial outcomes.

The methodological difficulties in evaluating the service and demonstrating cost-effectiveness were widely acknowledged in Site B. There was a preference for collecting quantitative data (evidence of cost savings), which ultimately failed to capture the impact LAC had on people’s lives. The lack of interest in individual ‘success’ stories and demand for numerical data were seen as instrumental in the project’s demise:

“I think it’s really difficult to measure it in terms of saving and I think … big managers focused on savings and want the numbers. Whereas something like this I think you can only measure with the stories, I think that’s where as I said the frustration comes in because … a lot of resource was invested in it and a lot of time and then literally to pull something within two years when it’s not that it’s because it’s been a flop, I don’t believe that. If it had been like a flop it’s different isn’t it? You could say ‘Oh the pilot’s not worked’, but I don’t believe that that’s been the case.” (Site B, Local Area Coordinator)

Similar to Site A, stakeholders in Site B understood the significance of demonstrating impact in order to secure future sustainability, but LAC coordinators continued to struggle with how to demonstrate its positive impact in the way that seemed to be required by decision-makers. Coordinators deemed the lack of guidance they had received about how this could be done as a significant barrier:

“I’ve just found out recently that the care navigators have been decommissioned because they haven’t evidenced their cost effectiveness ….We can give fantastic examples of where we’ve connected people and where people’s lives have changed but people want hard figures, don’t they?” (Site B: Local Area Coordinator)

Unrealistic time-scales set for gathering meaningful evidence was identified as another barrier to collecting outcome data in Site B. The pressure to demonstrate cost-savings was evident at the time of the first stakeholder interviews, when LAC had only been in operation for around nine months. The coordinators in Site B felt that the time-scale for being expected to generate evidence of progress and cost-effectiveness was too short, especially as the first three months of their employment were devoted to getting to know their communities.

“So although LAC was clearly established as long term, something that would start slowly and take time, I feel that we’re being challenged to show that we’re making immediate cashable savings and I don’t think that’s the bases on which we were set up.” (Site B: Local Area Coordinator)

In Site B, LAC was unable to demonstrate cost savings in such a short period of time and without this evidence the LA would not commit to it financially. Ultimately, LAC was seen by senior managers as ‘not giving a good return on investment’. With hindsight, several stakeholders commented that insufficient thought had been given to the need for partnership and commitment at a strategic level to plan service provision, maximise co-operation, avoid duplication and ensure that LAC (and other initiatives, such as care navigation) would be sustainable.

Despite the difficulties each site experienced in producing ‘hard’ outcomes data, stakeholders, community members and the coordinators themselves were able to provide many examples of how their work had improved the lives of individuals and their families. This was mostly achieved through enabling individuals and communities to ‘help themselves’. Key outcomes identified included: the development of personal skills, such as in managing finances and self-care; building of social networks which reduced isolation and increased community capacity; and increasing individual and community morale. These successes were attributed to the continuity of relationships, building trust, and engaging with people in personal and meaningful ways:

“It’s on a much more personal level. You’re talking about people and valuing people. That is absolutely the core of what LAC is, other professionals focus on paperwork, doing the assessments, care and support plans.” (Site B: Local Area Coordinator)

### Collaboration with other agencies and communities

Local Area Coordinators in both sites were faithful to the LAC model in terms of rooting themselves firmly in their local communities. This was seen as an important enabler of engaging key community ‘actors’ and resources to develop community capacity. For example, a Local Area Coordinator in Site A identified people who struggled with healthy eating and lacked opportunities to eat socially. The local authority worked with a local organisation willing to offer a venue and established a cook-eat group. In Site B, the support of local organisations was enlisted to provide resources to help set up a Junk Food project and a community café in one of the more deprived areas of the Borough. The projects in both Sites were instigated by the ideas and needs of local people. They were sustainable in the longer-term by the securing of local funding and through staffing by volunteers who gained skills and qualifications in necessary areas such as food hygiene and first aid.

The sites also shared some similar tensions in their relationships with other organisations and services. LAC was being implemented at a time of austerity and service cut-backs. Coordinators in both sites commented on the complexity of the difficulties faced by the people they worked with, particularly in relation to adverse social circumstances and mental health. Coordinators felt they were having to ‘fill the gap’ left by the withdrawal of other services and that this risked undermining their unique and distinctive role.

“They bat it back to us to pick it up and it’s not really a role for us, but no-one else is going to pick it up.” (Site A: Local Area Coordinator)

The most problematic relationship with other agencies in both sites seemed to be with social work teams. In both boroughs, Local Area Coordinators were paid the same salary grade as entry level social workers and this generated some resentment amongst social workers who saw the coordinators as unqualified and having a less challenging role. There was also uncertainty in both sites about the boundaries of their respective roles and responsibilities. In Site A, coordinators feared that a reorganisation of social work services to community teams might duplicate and undermine their roles and threaten their long-term sustainability. Senior managers were deliberating whether to amalgamate LAC with community social work, which risked diluting it, or retain LAC as a distinct role, which then raised questions about the interface between the LAC and community social work roles. In Site B, a decision was made to discontinue LAC as a distinct service but to embed elements of its principles and practices in new community development teams.

Despite some similarities, there were significant differences between the two sites in their relationships with their local communities and other organisations. Much of this stemmed from the historical context of these relationships. In Site A, a history of strong collaboration between agencies and sectors was described as the ‘bedrock’ of the implementation of LAC and this carried through into day-to-day management and practice. A multi-agency steering group with wide representation from agencies including health, the voluntary sector, housing, HealthWatch, public health, fire and rescue services and the police, generated knowledge, interest and commitment on the part of these services towards LAC.

“The fact that we kind of built for years on a strong partnership so we’ve been very lucky in having the same personnel in place across health, social care, voluntary community sector for a number of years now, I think that, and all seemingly thinking in the same direction. I think that’s really, really made a big difference.” (Site A: Local Authority Manager)

Local Area Coordinators developed collaborative relationships with other local agencies, who understood their role and could refer people to them. Site B lacked this strong foundation of multi-agency collaboration and the coordinators struggled to convey a clear sense of their role to others, including both community members and other professionals and organisations. The restriction of the implementation of LAC to only five out of seventeen wards made it difficult for practitioners and organisations whose remit was Borough-wide to know whether LAC was operating in a particular area. Although more could have been done to promote the service, there was reluctance to do this when it seemed increasingly unlikely that LAC would continue.

Site A worked hard with the voluntary and community sector to ensure that LAC was seen as a complement to their activities rather than a duplication and threat. This helped with practical implementation on the ground but also provided another important set of allies to support continuation of funding. In Site B, LAC appeared more marginal to other stakeholders and activities, was seen to duplicate with other initiatives. It was particularly seen as overlapping with care navigation funded through health. Coordinators had to spend considerable time understanding and explaining the differences and connections between the two supports. Although there was a similar issue in Site A where the clinical commissioning group had introduced social prescribing, the coordinators were able to forge a more distinct ‘doing’ role, beyond the signposting undertaken by the social prescribers:

“I think we understand that signposting is not going to be achievable, so we try to walk alongside them so that they do access, they do get from A to B. And that’s where we get a lot of our positive outcomes, the real complex people that are just not able to do things independently at that time.” (Site A: Local Area Coordinator)

In Site B, inter-agency collaboration was undermined by the sense of competition that was fuelled by service cutbacks and threats to funding. Some local organisations who had faced reduction or withdrawal of funding for their own service commented that their organisation either provided or could have offered the same service delivered through LAC. Examples were given of over-lapping roles and duplication and, sometimes, a view that the coordinators lacked the expertise for some of the work they were undertaking.

## Discussion

One of the main motivations for pursuing integrated care is to overcome service fragmentation and the associated consequences of service users falling between the cracks of care [42,43]. The evidence presented in this paper makes the case for LAC’s potential as a progressive way of addressing such fragmentation. The model’s underlying approach in addressing health and social care needs for individuals and communities by bridging the gap between individual resources, community assets and statutory services can be seen as encompassing the key facets of person-centred integrated care. At ground level across the two sites, this was achieved through the coordinators taking a holistic approach, prioritising the importance of personal relationships and recognising the power of engaging people in meaningful ways, with positive outcomes for service users. Despite these similarities, Site A was able to more successfully implement LAC through establishing secure funding from the outset, widespread and sustained senior manager and political support, and engagement of partners and community organisations through steering groups and networking.

Research has highlighted that successful implementation depends on an agreed vision of the role of the coordinators and associated support for this vision from their employing organisations and the wider system [35]. For example within the Australian NDIS scheme the community development role of LAC has been compromised through recent emphasis on meeting performance targets for completion of individual support plans dominating their capacity [44]. Other implementation factors found in the United Kingdom and elsewhere include the area’s ability to recruit and retain coordinators, the size of the geographic area and therefore population for which the coordinators are responsible, the degree of understanding and support from operational and senior managers, and the communication with the relevant population about the nature and potential benefits of this approach [45]. Resistance from existing services and professionals has also been highlighted elsewhere as an issue through misunderstanding of the role of coordinators and concerns about their responsibilities being replicated or replaced [46].

Organisational issues were determining factors in the overall outcome of LAC within the sites [47,48]. Successful implementation of LAC appeared to be reliant on an explicit long-term commitment to the model (i.e. ‘we will make this work’), versus a more measured pilot application (i.e. ‘we will see if this can work’). This questions the common logic that short-term pilots are the most effective approach to selecting the most effective intervention [49]. Rather, it may be better to invest time up front to select the most appropriate interventions through existing evidence and practice experience, and then commit resources to implementing the selected interventions thoroughly over a realistic time-period. Such an approach helps to avoid local confusion, reflects the reality that implementation (and therefore impact) take many years, and avoids disillusionment when pilot programmes which are valued within communities are withdrawn at an early point. Ensuring that at least part of the funding for a new approach is drawn from mainstream finances provides further reassurance and stability; if drawn from multiple partners, this consolidates their interest and long-term engagement [50].

Alongside organisational issues, this study reinforces the role of people and culture to implementation [21,51]. Leaders in senior roles are instrumental in developing the vision, providing momentum, and maintaining these in the long term as contexts and dynamics change. Practice leaders are central to establishing local relationships and influence, and in turning the strategic promise into one of improved wellbeing for individuals and families [52]. Even when a model has clear principles and structure, community-based integration requires local interpretation and the facilitation of skills of ‘social entrepreneurism’ [53]. New integrated care approaches are not introduced in a vacuum, and there is a strong likelihood that existing professions and services may see them as a criticism of their own practice and a threat to their existence [54,55]. Whilst such sensitivities should not prevent new approaches, listening to such concerns can provide insights into how duplication may be avoided and reduce resistance based on inadequate information and ill-founded rumours. For those within services that will be replaced, they will at least understand the rationale behind this decision. Lastly, basing implementation on common values and objectives provides a sound bedrock for supporting partnerships and integrated working across organisational boundaries.

## Conclusion

This study suggests that LAC can be an effective means to enabling asset-based integrated care. Beyond consideration of the adoption of this model per se, the main learning for other systems is that community based integration should not only seek to connect people with existing community assets but also have the capacity and skills to facilitate the development of new resources to respond to identified needs.

The experiences of these two sites reflect the common complexity of implementation and the many factors that can lead to a promising intervention not being well received in a local context [56]. The findings reveal the detailed learning about the implementation of asset based approaches to integrated care, through what was successful and challenging in both sites, whilst highlighting the differences between their approaches to implementation of the same intervention.

Unlike many such studies, it also confirms that implementation challenges can be overcome if there is sufficient attention to both organisational and people-based issues. These include: maintenance of a common vision of what an intervention will achieve and how it will work in practice; continual engagement with the political and organisational leaders of influence; positively addressing the anxieties of existing services and professions; and working with community groups. This also includes learning about the importance of stakeholder relationships within a wider system that will influence the deployment of an intervention, and how these can be engaged with proactively to increase successful implementation.

Finally, it highlights that further work must be done to address the methodological difficulties of evaluating outcomes within such initiatives. For example, understanding what outcomes are likely to be realised by such interventions and when by, and the methodologies that will provide an appropriate assessment of the success of the interventions.

Without robust and timely evidence, the potential contribution of such community-based approaches may not be recognised by funders and policy makers.
